# Drug Reaction with Eosinophilia and Systemic Symptoms (DReSS)/Drug-Induced Hypersensitivity Syndrome (DiHS)—Readdressing the DReSS

**DOI:** 10.3390/biomedicines10050999

**Published:** 2022-04-26

**Authors:** Hannah Stirton, Neil H. Shear, Roni P. Dodiuk-Gad

**Affiliations:** 1 Section of Dermatology, Department of Medicine, University of Manitoba, Winnipeg, MB R2M 3Y8, Canada; stirtohe@myumanitoba.ca; 2 Division of Dermatology, Department of Medicine, University of Toronto, Toronto, ON M5S 1A1, Canada; 3 Temerty Department of Medicine, University of Toronto, Toronto, ON M5S 1A1, Canada; neil.shear@sunnybrook.ca; 4 Emek Medical Centre, Afula 1855701, Israel; 5 Bruce Rappaport Faculty of Medicine, Technion Institute of Technology, Haifa 3525433, Israel

**Keywords:** drug reaction, drug-induced hypersensitivity syndrome, drug reaction with eosinophilia and systemic symptoms, delayed hypersensitivity, regulatory T cells, human leukocyte antigen (HLA), viral reactivation

## Abstract

Drug reaction with eosinophilia and systemic symptoms (DReSS), also known as drug-induced hypersensitivity syndrome (DiHS), is a severe, systemic, T cell mediated drug reaction with combinations of cutaneous, hematologic, and internal organ involvement. Pathogenesis of DReSS is multi-factorial, involving drug-exposure, genetic predisposition through specific human leukocyte antigen (HLA) alleles and metabolism defects, viral reactivation, and immune dysregulation. Clinical features of this condition are delayed, stepwise, and heterogenous, making this syndrome challenging to recognize and diagnose. Two sets of validated diagnostic criteria exist that can be employed to diagnose DReSS/DiHS. Methods to improve early recognition of DReSS and predict disease severity has been a recent area of research focus. In vitro and in vivo tests can be employed to confirm the diagnosis and help identify culprit drugs. The mainstay treatment of DReSS is prompt withdrawal of the culprit drug, supportive treatment, and immunosuppression depending on the severity of disease. We present a comprehensive review on the most recent research and literature on DReSS, with emphasis on pathogenesis, clinical features, diagnosis, confirmatory testing modalities, and treatment. Additionally, this summary aims to highlight the differing viewpoints on this severe disease and broaden our perspective on the condition known as DReSS.

## 1. Introduction

Drug reaction with eosinophilia and systemic symptoms (DReSS) or drug-induced hypersensitivity syndrome (DiHS) is a severe, idiosyncratic, T-cell mediated hypersensitivity reaction characterized by varied combinations of skin eruption, fever, facial edema, lymphadenopathy, hematological abnormalities, and visceral involvement [1]. Compared to other severe cutaneous adverse drug reactions (SCARs) such as Stevens-Johnson syndrome (SJS), toxic epidermal necrolysis (TEN) and acute generalized exanthematous pustulosis (AGEP), DReSS has a more heterogenous clinical presentation making it challenging to diagnose.

While the triad of drug induced fever, rash, and eosinophilia has been recognized since the 1930s, the term DReSS is more recent [1]. Because eosinophilia is not always present and other hematological abnormalities may occur, many different titles have been used to define this syndrome. Originally recognized as anticonvulsant hypersensitivity syndrome, other terms have been proposed over time such as drug-induced pseudolymphoma, drug-induced delayed multiorgan hypersensitivity syndrome (DIDMOHS), and hypersensitivity syndrome [2,3]. In 1996 Bocquet et al. [4] proposed the term DRESS (Drug Rash with Eosinophilia and Systemic Symptoms) to encompass these similar reactions and differentiate them from other severe drug reactions without eosinophilia. The word “rash” in DReSS was subsequently changed to “reaction” due to its diverse cutaneous manifestations. The lowercase e is often used, as in this review, to denote that eosinophilia is not always present and other hematological abnormalities may be seen [1,5]. The term DReSS became internationally recognized and gained popularity in multiple countries, although it is not used everywhere [3,4]. The Japanese investigators Shiohara and Kano [6] coined the term “Drug-induced hypersensitivity syndrome” (DiHS) to describe a version of DReSS requiring the presence of viral reactivation. Today, although still somewhat controversial, the terms DReSS and DiHS are often used interchangeably to describe the same syndrome. More recently, some have suggested that the “D” in DReSS should be re-examined as vaccines and biologics have also been shown to trigger DReSS [7,8,9]. Based on the diversity of the clinical features and non-drug precipitants of DReSS, a further change in nomenclature may be required in the future to reflect this heterogeneity.

Over the last decade there has been significant improvement in the understanding of the different clinical presentations of DReSS, its prognostic factors, underlying pathophysiology, and methods to treat and prevent future reactions through intervention and screening. This review not only aims to summarize recent updates on DReSS, but also to highlight the differing viewpoints on this severe disease, broaden the collective perspective on what is understood to be DReSS, and reconsider a more inclusive designation.

## 2. Literature Search Methods

A literature review of DReSS/DiHS was carried out by searching PubMed/MEDLINE and the Cochrane Database of Systematic Reviews between January 1950 and March 2022. Emphasis was placed on the most recent publications, with at least 50% of studies cited as published within the last 5 years. Search terms were “DRESS”, “drug reaction with eosinophilia and systemic symptoms”, “drug rash with eosinophilia and systemic symptoms”, “drug hypersensitivity and eosinophilia”, “drug-induced hypersensitivity syndrome” and “viral reactivation”. Among this group of articles, global opinion leaders on this topic selected the most important papers to be included in this review.

## 3. Epidemiology

Due to its varied clinical presentation and complex nomenclature, there is limited epidemiological evidence on DReSS. The estimated risk at first or second prescription of aromatic antiepileptic is approximately 1:1000–1:10,000, although this is highly dependent on the ethnic background of the individual [10]. Incidence rates range from 3.89 per 10,000 inpatients in Spain, to 0.9 per 100,000 people in a West Indian population [11,12]. Prevalence estimates include 2.18 per 100,000 in the US and 9.63 cases per 100,000 inpatients in Thailand [13,14].

Gouveia et al. [15] found that DReSS was the most frequent cutaneous adverse drug reaction in patients admitted to hospital compared to other SCARs. While DReSS does occur in children, it is predominantly seen in adults with a mean age of onset between 40 and 60 years [14,15,16,17]. In the 2013 RegiSCAR study they also found female patients to be significantly younger than their male counterparts [15]. While several studies have found a slight female predominance in DReSS, many more have not replicated this finding [15,16]. There is significant association between ethnic background and DReSS, with an abundance of research showing certain human leukocyte antigen (HLA) alleles being a strong risk factor with exposure to certain drugs [18].

The most common comorbidities of DReSS include epilepsy, HIV, hypertension, diabetes, and hyperuricemia [14,15,16]. These co-occurring disorders are likely related to the culprit drug rather than an intrinsic predisposition to DReSS [19].

## 4. Pathogenesis

**Figure 1:**
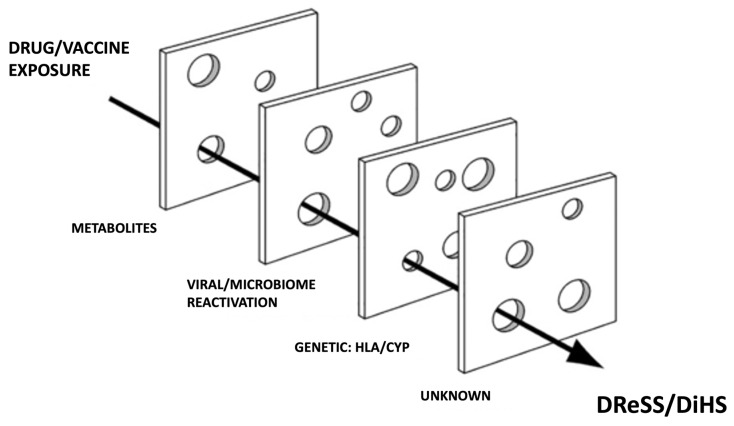
“Swiss cheese” risk model of DReSS/DiHS. *CYP* cytochrome P450, *HLA* human leukocyte antigen, *DReSS* drug reaction with eosinophilia and systemic symptoms, *DiHS* drug induced hypersensitivity syndrome.

Significant advances in the understanding of DReSS pathogenesis have been made recently. DReSS is a severe, idiosyncratic, T cell mediated drug reaction, classified as a delayed type IVb, and sometimes IVc, hypersensitivity reaction [1]. It is assumed that DReSS is the result of a complex interaction between drug (or vaccine or biologic) exposure, genetic predisposition, and viral reactivation [1]. Why some develop this condition while others do not, despite the same exposure, is thought to be a result of the cumulative effect of aligned risks that can be likened to a “Swiss cheese” model as illustrated in Figure 1. There are likely other risk factors and predisposing conditions leading to this disease still unknown that need to be accounted for in this model.

### 4.1. Viral Reactivation

The relationship between viral reactivation and DReSS has been studied extensively [20]. Despite this, there is still much controversy surrounding this topic. Even with the abundance of research on this matter, questions regarding its clinical relevance, role as a causative factor vs. a complication, and validity of viral testing techniques, remain mostly unanswered.

Historically, human herpes virus-6 (HHV-6) has been most associated with DReSS, although other human herpes viruses have been reported including HHV-7, cytomegalovirus (CMV), Epstein-Barr virus (EBV), and herpes simplex virus (HSV) [21,22]. There are multiple studies demonstrating a connection between DReSS and viral reactivation, so much so that the diagnosis of DiHS by the Japanese consensus group requires evidence of HHV-6 infection [23,24]. However, viral reactivation is less reported in cases outside of Japan, and some have suggested the incidence may be over-estimated owing to testing techniques [16,25].

The incidence rates of HHV-6 reactivation are highly variable across studies, ranging from 36% in the multi-national RegiSCAR study, to 62% in a Japanese-specific population [16,26]. A 2013 systematic review found that less than 50% of studies tested for viral reactivation, but when performed, the majority showed evidence of reactivation with an HHV-6 reactivation rate of 80% [27]. A 2010 prospective study by Picard et al. [28] found 76% of DReSS patients had evidence of viral reactivation with some combination of EBV, HHV-6 and/or HHV-7. Rates of viral reactivation in the healthy control group, comparatively, were 0%.

Viral reactivation typically occurs 2–4 weeks after symptom onset and has been associated with longer disease duration, flares, and more severe outcomes [21,23,24,26,29,30,31,32]. Specifically, patients with viral reactivation were found to have a longer disease duration, an increased number of relapses, and increased rates of lymphadenopathy, hepatitis, renal failure, encephalitis, myocarditis, severe lymphopenia, and death, compared to DReSS patients with no evidence of viral reactivation [26,31,32]. diKano et al. [33] found patients can experience sequential reactivation of multiple viruses that may explain the symptom relapses seen in DReSS despite drug withdrawal. This finding has been compared to the sequential viral reactivation seen in acute graft versus host disease (GVHD) [33].

Multiple mechanisms have been proposed to explain why viral reactivation occurs in DReSS. In the acute phase, a relatively immunocompromised profile is seen with the rapid expansion of immune suppressive T regulatory cells (specifically CD4+CD25+FoxP3+) and concomitant decreases in immunoglobulins and peripheral B-cells [33,34] This clonal expansion may reduce the function of anti-viral T lymphocytes leading to viral reactivation. Others have suggested that viral reactivation may be due to immunosuppression from corticosteroid treatment [35]. However, Tohyama et al. [26] showed HHV-6 reactivation occurred more frequently in patients not treated with steroids compared to high-dose steroid groups [29]. While high-dose pulsed steroids have been associated with CMV reactivation in other conditions, Mizukawa et al. [17] suggest that the rapid dose reduction in steroids may induce a rapid recovery of immune response that could contribute to the development of CMV reactivation and symptom worsening. Indeed, several studies have shown the steroid tapering phase to be a high-risk time for symptom recurrence [24,31,35]. Flares during the steroid tapering are theorized to either be secondary to the underlying inflammation of DReSS recurring, or possibly, reactivation of pre-existent but clinically undetectable viruses [36]. Shiohara et al. liken the latter phenomenon to the immune reconstitution syndrome seen in HIV [36]. Some authors have suggested that if an immune reconstitution-like syndrome is occurring in DReSS, it may indicate that viral infection is playing a more causative role in DReSS despite being undetectable in patients at initial presentation owed to robust host immune responses [17,36,37].

Recently, there has been interest in the idea that drug-reactive DReSS-inducing T cells are virus-specific memory T cells [38]. In other words, T cells previously generated in response to a viral infection are stimulated upon presentation with HLA-drug complexes and mistakenly reactivate [38] This is supported by evidence from Picard et al. [28] showing the expansion of EBV-specific CD8+ T lymphocytes in blood, skin, liver, and lungs of DReSS patients. Furthermore, in vitro studies showed HIV-specific T cells cross-reacted with HLA-B*57:01-positive cells in the presence of abacavir [39]. In a case report by Kim et al. [40], single cell RNA sequencing was used to examine the aberrant immune response in a patient with persistent DReSS from Trimethoprim-Sulfamethoxazole (TMP-SMX). Not only did they find the activation of the Janus kinase–signal transducer and activator of transcription (JAK-STAT) pathway, leading to successful treatment with the JAK-inhibitor tofacitinib, but they also found HHV-6b DNA was enriched in central memory CD4+ T cells. Impressively, in vitro studies demonstrated anti-viral medications targeting HHV6b were able to suppress TMP/SMX-induced CD4^+^ T cell proliferation in dose-dependent manners [40].

In summary, there is no global consensus as to if, or how, viral reactivation is associated with DReSS. As to whether it is one of the many contributing risk factors in the development of DReSS, a consequence of the drug-induced immune dysregulation, or a side-effect of the immunosuppression used in treating DReSS, this is still unknown [1,25,38,41].

### 4.2. Drugs

The most common DReSS-inducing drugs are anticonvulsants, allopurinol, sulfonamides, and antibiotics [14,16,42]. In the RegiSCAR study, aromatic anti-epileptics made up 35% of all drug-triggers, with carbamazepine alone making up 27% in one systematic review [16,27]. Carbamazepine is the most common cause of DReSS both overall and within the anticonvulsant grouping, with lamotrigine, phenytoin and phenobarbital also frequently reported [16,17,27,43,44]. Allopurinol has been the attributable drug in 11–18% of DReSS cases [16,27]. Sulfonamides are the probable causative drugs in around 12% of cases, with sulfasalazine often making up more than half of that grouping, although TMP-SMX and dapsone are also reported [16,27,42]. Non-antibiotic sulfa-drugs like furosemide are less associated with DReSS, but case reports do exist [42,45]. A systematic review by Sharifzadeh et al. [42] found anti-tuberculosis medications made up 42% of all antibiotic-related DReSS cases, with rifampin being the most common offending agent. Vancomycin was the second most common antibiotic comprising 18% of antibiotic related cases [42]. In the pediatric population the most common drugs are antiepileptics and antibiotics (vancomycin followed by TMP-SMX) [19]. A comprehensive list of drugs associated with DReSS are listed in Table 1. It is important to note that more recently vaccines and biologics have been shown to be capable of triggering DReSS [7,8,9].

### 4.3. Immune Changes

DReSS is characterized by a variety of hematological abnormalities including leukocytosis, atypical lymphocytosis, and eosinophilia [49]. Furthermore, a heterogenous profile of cytokines and chemokines have been found in DReSS [50]. While eosinophilia is not universally present, a Th2-type response can be seen with eosinophil-associated cytokines such as IL-4, Il-5 and IL-13 [50,51,52]. Serum thymus and activation-regulated chemokine (TARC/CCL17), commonly associated with the Th2 response, may also be elevated and possibly correlate with disease activity [53,54]. Specifically, TARC/CCL17 recruits Th2-polarized T lymphocytes into local inflammation sites. Other cytokines reported to be elevated in DReSS include IFN-γ, TNF-α, IL-2, IL-6, and granulysin [28,50,55,56,57].

In a small study by Nishio et al. [55], CD4+ cells increased early on in DReSS and decreased thereafter. They also found CD8+ T cells and Th1-cells increased later in disease, often concomitantly with disease flare, worsening hepatitis, and rises in anti-HHV-6 antibodies. These authors propose that CD4+ and CD8+ T cells might respond to causative drug and virus-infected cells, respectively. This trend in CD4+ and CD8+ T cell fluctuation has been replicated by Shiohara et al. [36].

Several studies have shown a dramatic expansion of the immune-suppressing CD4+CD25+FoxP3+ Treg cells in the early stages of DReSS, both in peripheral blood samples and in cutaneous lesions [34,58,59]. Skin homing Tregs are postulated to limit the severity of skin disease in DReSS through their suppressive effect on cytotoxic T cells [32]. While skin lesion severity may be limited through this mechanism, this expanded population might serve to prevent the activation and expansion of antiviral T cells, allowing latent herpesviruses to reactivate in an uncontrolled fashion [34]. At the resolution stage of DReSS, Tregs are found to be functionally impaired despite normal cell frequencies [34]. Some studies have shown an association between autoimmune conditions and the decreased suppressive function of Treg cells, which could explain a pathogenic mechanism for the development of autoimmune conditions seen in some DReSS patients [35,59,60,61].

### 4.4. Genetic Predisposition

HLA alleles are one of the most important risk factors in the development of DReSS. Importantly, certain high-risk alleles are present in some ethnicities more than others, making ethnic background an important predisposing factor to DReSS (see Table 2) [62]. Mechanistically, it is thought that the culprit drug interacts with a particular HLA to form a complex-hapten, which is then presented to naive T cells via the T cell receptor to stimulate an immune response [63].

One of the first examples of HLA association with drug hypersensitivity was in 2002 when the association between abacavir-induced hypersensitivity syndrome in HIV patients and HLA-B*57:01 was found [64,65]. With a negative predictive value of 100% in the PREDICT-1 trial and complete elimination of abacavir-hypersensitivity, screening for HLA-B*57:01 is now recommended by drug regulatory agencies in every patient prior to initiating treatment [64]. A 2005 study examining Han-Chinese patients found a strong association with HLA-B*58:01 and allopurinol-induced DReSS (among other SCARs), present in 100% of cases examined [66]. The 2012 American College of Rheumatology Guidelines recommend testing for the HLA-B*58:01 allele in selected subpopulations (individuals of Korean descent with stage 3 or worse chronic kidney disease and those of Han-Chinese or Thai descent) prior to the initiation of allopurinol.

Similarly, multiple studies have shown an association between HLA-A*31:01 and carbamazepine-induced DReSS, with positivity rates ranging from 37–67% among multiple ethnicities [67,68,69,70,71]. Per the Canadian Pharmacogenomics Network for Drug Safety [72], genetic testing for HLA-A*31:01 is recommended for all carbamazepine-naive patients before the initiation of therapy. While the link between A*31:01 and carbamazepine-induced DReSS may not be as strong as with other alleles, it is one of the most common alleles in most populations studied, making it an important screening tool to prevent DReSS [72]. A comprehensive table of the current known drug-HLA associations in DReSS are listed in Table 2.

Importantly, drug-HLA associations often have a high negative predictive value (NPV) and moderate-to-low positive predictive values (PPV). To date, the highest PPV known is around 50% in abacavir-induced DReSS [73]. In other words, the low PPV of HLA allele associations suggests that additional factors contribute to the onset of disease. Furthermore, allelic frequencies vary greatly between different ethnicities, making it important that recommendations not only be made based on the drug, but on ethnic origin as well. With the explosion of research on this topic, the list of DReSS related drug-HLA associations is expected to grow in the coming years, further contributing in the movement towards personalized medicine.

**Table 2:** DReSS-associated human leukocyte antigen (HLA) alleles according to drug and ethnicity.

Drug	HLA Allele	Ethnicity	References
Allopurinol	B*58:01	Han Chinese, Korean, Taiwanese, Thai	[66,67,74,75,76,77,78]
Carbamazepine	A*31:01	European, Chinese, Korean, Japanese	[68,69,70,71,79,80]
Dapsone	B*13:01	Chinese, Taiwanese, Thai	[81,82,83,84]
Salazosulfapyridine	B*13:01	Han Chinese	[85]
Phenytoin	A*24:02	European (Spanish)	[80]
	B*15:13	Malaysian	[86]
	B* 51:01	Thai	[87]
	C*14:02	Thai	[87]
Lamotrigine	B*51:01 and A*24:02	European (Spanish)	[80]
Piperacillin/tazobactam	B*62	UK caucasian	[88]
Vancomycin	A*32:01	North American	[89]
Abacavir *	B*57:01	European, African, North American	[64,65,73,90]
Nevirapine *	CW*04:01	Han Chinese, Thai, Malawian	[91,92,93,94]
	Cw*8/Cw*08-B*14	Italian, Japanese	[95,96]
	B* 35:05	Asian (Thai)	[92,97]
	B*35:01	Australian	[98]
	DRB1∗01:01	Australian	[97]
Raltegravir	B*53:01	African, Hispanic	[99]

* Do not completely meet criteria for DReSS syndrome.

Mutations in several drug detoxification enzymes have also been linked to DReSS. Aromatic anticonvulsants are metabolized by the cytochrome P450 (CYP-450) system to arene oxide metabolites, normally detoxified by epoxide hydrolase or glutathione transferase to inactive metabolites [100]. Through in vitro lymphocyte transformation assays Shear and Spielberg [101] showed evidence of epoxide hydrolase deficiency in anticonvulsant-induced DReSS patients [102]. The arene oxide intermediates may act as haptens to stimulate the immune response or bind to tissue macromolecules and cause cell damage directly.

Shear et al. [101] describe a similar phenomenon in sulfonamide-related DReSS, finding high incidence of slow N-acetylation status in these patients [101]. Because of the relative N-acetyltransferase deficiency, the CYP-450 oxidative pathway is favored, leading to toxic levels of hydroxylamine metabolites capable of causing cell damage and immune activation. In a study examining patients from Taiwan, Japan and Malaysia, the CYP2C9*3 gene variant (known to significantly reduce phenytoin clearance) was significantly associated with phenytoin-related SCARs including DReSS [103].

As research has shown increased risk of allopurinol-related DReSS in HLA-B*58:01 positive patients with reduced kidney function, it is possible that elevated levels of drug or toxic drug metabolites may interact with specific HLA to further increase the risk of DReSS development [77,104]. Indeed, it does not seem that the presence of drug-specific HLA-alleles or drug metabolism polymorphisms alone are enough to trigger DReSS, but taken together may function synergistically to elevate risk.

## 5. Clinical Features 

DReSS is characterized by stepwise multi-organ involvement that may include skin, hematological systems, and solid organs. Most commonly it begins with a flu-like prodrome of malaise, pharyngitis, fever, and lymphadenopathy [27]. The progression of signs and symptoms can be slow and in varied combinations, but most studies report fever in most patients (between 75–100%) [1,16,28,43,105]. Fever typically precedes the cutaneous eruption by several days.

Compared to other SCARs, the lag time between drug exposure and symptom onset is more delayed, typically between 2–8 weeks (although longer and shorter times have been reported) [16,27]. Upon re-exposure to the culprit drug symptoms can develop in hours to days [16,27]. Lag time may also differ depending on the drug [16]. For example, antiepileptics and allopurinol tend to have longer latency periods compared to antibiotics or radiocontrast media, which have been shown to have lag times less than 14 days from exposure [106,107,108]. Longer latency periods may correlate with more severe disease [105].

The cutaneous manifestations of DReSS are diverse. Typically, more than 50% of total body surface area (BSA) is involved [16,27,109]. The most common morphologies reported are monomorphic maculopapular/morbilliform, urticated papular, and exfoliative erythroderma [16]. Shiohara et al. [6] describe the rash starting as patchy erythematous macules, pustular, target-like or eczema-like lesions, that can become purpuric and confluent over time. An erythema multiforme (EM)-like eruption with atypical targetoid lesions has also been described, which was associated with more severe hepatic involvement in one study [110]. Distribution is typically symmetric, often starting on the face, upper trunk and upper extremities and then spreading to the lower extremities [6]. Cutaneous manifestations are polymorphic in around 85% of cases, which can include secondary features such as pustules, purpura, vesicles, bullae and cheilitis [4,16,43]. Facial edema is also characteristic of DReSS (reported in up to 76% of cases), and may be a distinguishing feature from more mild forms of DReSS or maculopapular eruption (MPE) [6,15,16,105]. Mucosal involvement can be seen in up to 56% of patients, however it is typically mild and non-hemorrhagic, distinguishing it from SJS/TEN [15,16,43]. It was reported that in pediatric DReSS, children are more likely to have a morbilliform exanthem, fever, and lymphadenopathy, but less likely to have facial edema [19,111].

There are a range of hematological abnormalities seen in DReSS. Hypereosinophilia is the most common finding, present in 52–92% of patients across multiple studies (although the majority show greater than 80%) [14,15,16,17,27,43,105,107]. Eosinophil counts are often dramatically increased with an average eosinophil count of 3.5 (×10^9^ L^−1^) [27]. Leukocytosis with early neutrophilia and delayed monocytosis is the next most common, followed by atypical lymphocytosis in 27–67% of patients [3,16,27,28,43,107]. Other less frequent findings include lymphopenia, leukopenia, thrombocytopenia, thrombocytosis, and pancytopenia, which are associated with a more severe prognosis [15,16,43,107].

Liver injury is the most common visceral manifestation in DReSS, seen in up to 97% of cases [16,27,28,112,113]. Elevated liver enzymes (cholestatic, mixed and hepatocellular have all been reported) is the most common finding; however, liver failure with or without subsequent transplant does occur [15]. The hepatitis is typically anicteric and liver enzyme elevation may take months to completely resolve [37].

The next most involved organ is the kidney [1,16,114]. Renal involvement in DReSS ranges from mild acute kidney injury (AKI) to severe interstitial nephritis, sometimes resulting in permanent end-stage renal disease. Elderly patients, allopurinol-associated DReSS, and those with pre-existing kidney disease are at the highest risk of renal impairment [113]. The lung is the third most frequently impaired organ, with interstitial pneumonitis being the most common manifestation [114]. Minocycline-associated DReSS has been associated with a higher incidence of pneumonitis [109]. Cardiac involvement in DReSS is becoming more frequently recognized, typically presenting as myo- or pericarditis [115]. Cardiac disease is often delayed in DReSS, occurring on average 70 days after the initial symptoms. The most common signs and symptoms of cardiac DReSS are dyspnea, cardiogenic shock, chest pain, and tachycardia.

More incidentally there have been reports of the following: pancreatitis, colitis, cholangitis, encephalitis/meningoencephalitis, hemophagocytic syndrome, and thyroiditis [16,27,28,113,116].

## 6. Diagnosis

Making a diagnosis of DReSS can be challenging due to its delayed-onset, stepwise presentation, and variable clinical features. In 2006 the Japanese consensus group put forth their criteria for the diagnosis of DiHS (Table 3), followed by the RegiSCAR group in 2007 proposing their scoring system for DReSS (Table 4) [5,23]. An important distinction between the two scoring systems is the requirement of HHV-6 reactivation for typical DiHS. While the RegiSCAR score is more frequently used, particularly in North America and Europe, both scoring systems evaluate similar criteria and play important roles in the diagnosis of this condition.

One drawback of both sets of criteria is their inability to allow for an early diagnosis of DReSS. Recently, Choudary et al. [117] created a predictive model that incorporates baseline measures of % BSA, eosinophil count, and CRP. They found a 96% sensitivity and a 100% specificity at predicting DReSS compared to MPE. Interestingly, they also found the baseline serum TARC levels (with a cut-off of 613.25 pg/mL) to have excellent diagnostic performance with an 88% sensitivity and a 100% specificity in predicting DRESS on its own.

### 6.1. DReSS Minor/DReSS Major

There has been recent interest in further defining the spectrum of DReSS severity. Some have proposed separating into categories of simple MPE (maculopapular eruption), DReSS minor (other terms include mini-DReSS, MPE/DReSS overlap, and systemic MPE), and DReSS or “DReSS major” [15,105,118]. Determining predictive factors of disease severity and the clinical features that may help delineate between these phenotypes has also been investigated. Momen et al. [105] defined DReSS major as a RegiSCAR score of ≥4 (with DReSS minor as 1–3) and found that DReSS major patients experienced significantly more facial edema, higher liver enzyme elevations, and required longer courses of immunosuppression than DReSS minor patients. The authors of this study concluded that DReSS minor should therefore be distinguished as a milder form of the disease from DReSS major, and that facial edema can help discriminate between major and minor forms. Skowron et al. [118] classified “systemic MPE” (sME) as MPE with the presence of fever or organ involvement without meeting DReSS criteria. Similar to the results by Momen et al. [105], they found facial edema, hepatic involvement, fever, and eosinophilia were significantly more frequent in DReSS compared to sMPE. Gouveia et al. [15] defined MPE/DReSS overlap as an exanthem plus one or two systemic symptoms or laboratory abnormalities, but a RegiSCAR score < 4. Somewhat contrary to the previous studies, they found that while MPE/DReSS overlap presented with fewer features of DReSS, these patients showed similar disease severity and length of hospitalization. The authors therefore concluded that this overlap syndrome should be considered within the *same* category as DReSS with regards to management and follow-up.

As evidenced by these three studies, there is still much controversy regarding the milder forms of DReSS and how they should be classified and managed moving forward. What can be agreed upon is that DReSS clearly exists on a spectrum, with mild cases showing fewer systemic signs and symptoms, and patients at the opposite end potentially developing life-threatening organ dysfunction [15]. This recent focus on the variability in DReSS presentation highlights the collective shift towards a broadened, deeper, and more inclusive understanding of DReSS.

### 6.2. Causality Assessment and Confirmatory Testing

There is no clear consensus on the ideal method of determining the causative drug in DReSS, although several systems have been proposed. The Spanish Guidelines for Diagnosis, Management, Treatment, and Prevention of DRESS Syndrome suggest using the Algorithm of the Spanish Pharmacovigilance System, which considers chronology (suggestive if the drug was initiated less than 6 months previously and stopped less than 14 days before the index day), known drug association in the literature, an improvement with drug withdrawal, and the positive re-challenge effect [47]. Kardaun et al. [16] suggest excluding a drug from consideration if the drug was taken for more than 3 months, had been stopped more than 2 weeks before the index day, or had been initiated less than 3 days before the probable index day.

There are two in vitro tests, the lymphocyte transformation test (LTT) and the enzyme-linked immunospot (ELISpot) assay, that can be used to help identify the culprit drug. The LTT measures T cell proliferation, specifically H-thymidine, in response to a drug after incubation [119]. While laboratories have reached a consensus regarding the protocol and cut-offs for positivity, there are no standard values for each drug [120]. Sensitivity and specificity ranges from 58–73% and 82–95%, respectively, with higher values for anticonvulsants, antituberculosis drugs and B-lactams [121,122]. A negative result is not overly helpful owing to its lower sensitivity, but a positive result reflects specific sensitization to the test drug that can help support the diagnosis and determine the culprit agent if the patient took several drugs. The ELISpot determines the number of cells that release relevant cytokines and cytotoxic markers after their activation by the culprit drug [119]. Positive ELISpot assays for IFN-γ production have been reported in DRESS, however no consensus exists on the criteria for positivity, and it is less frequently employed [47]. Neither test should be performed until at least 4–8 weeks after recovery and 4 weeks after stopping steroids.

In-vivo confirmatory test options include patch testing, skin pricks, delayed intradermal and controlled re-exposure. Patch testing was found to be positive between 57–64% of DReSS cases, with significant variability depending on the drug [123,124]. While significantly less studied, delayed skin prick and intradermal testing has been shown to have positive results in DReSS and is suggested by the Spanish guidelines if initial patch testing is negative [47]. Despite the relatively good safety profile of patch and intradermal testing, both have been known to cause the recurrence of DReSS symptoms in some cases [124]. Controlled re-exposure is absolutely contraindicated unless special circumstances exist, such as DReSS related to anti-tuberculosis medications and beta-lactams [47]. In one study of exclusively anti-tuberculosis drug related DReSS, reintroduction led to a relapse of symptoms in seven out of the 13 patients, with three meeting criteria for recurrent DReSS [125].

At present, the LTT is the best documented assay for the in vitro diagnosis of DReSS and the Spanish guidelines strongly recommend performing the LTT and/or the ELISPOT to confirm the diagnosis [47]. The international consensus of in vitro methods for the diagnosis of drug hypersensitivity reactions by the European Academy of Allergy and Clinical Immunology (EAACI) gave a grade C recommendation to both the LTT and ELISpot [119]. A comparison of skin tests and LTT confirms a higher sensitivity and specificity of LTT in DReSS syndrome, and both the Spanish guidelines and EAACI suggest performing in vitro tests prior to in-vivo tests. If in vitro testing is not available, patch testing may be considered first-line.

Overall, confirmatory testing is reasonable and may be advised in high-risk patients when the potential benefits outweigh the risks. This may include patients where there is diagnostic uncertainty, multiple possible causative agents, and when the suspected culprit drug is necessary for a critical health condition and no reasonable alternatives exist [47,119,125].

## 7. Histopathologic Findings

There is no single histopathological finding to characterize DReSS. In fact, one of the distinguishing features from non-drug dermatoses and MPE is multiple inflammatory patterns in a single biopsy [126]. Ortonne et al. [126] found an interface dermatitis was most common (74%), followed by eczematous (40%), EM-like (24%) and AGEP-like (20%). In a study of 27 DReSS patients, Walsh et al. [110] found that spongiotic dermatitis with a superficial perivascular lymphocytic infiltrate was the most frequent pattern (around 60% of biopsies). This same report also found patients with an EM-like cutaneous reaction were associated with the presence of necrotic keratinocytes and basal cell vacuolization, as well as more severe liver dysfunction. Chi et al. [127] found that the presence of severe dyskeratosis was significantly associated with the clinical severity of renal impairment. While epidermal necrosis is not the hallmark of DReSS as it is in SJS/TEN, a significant proportion of cases demonstrate epidermal damage, necrotic keratinocytes and severe dyskeratosis, which may help distinguish from MPE or less severe cases of DReSS [43,49,118,127].

Frequent epidermal findings include para- and/or orthokeratosis, dyskeratosis, lymphocyte exocytosis, spongiosis, necrotic keratinocytes, basal cell vacuolization, focal lichenoid interface dermatitis, and non-follicular spongiform pustules [15,43,49,118,126,127,128]. In the dermis, one of the most frequently reported findings is a predominantly lymphocytic infiltrate, although atypical lymphocytes, neutrophils, and eosinophils are invariably present [15,43,49,118,127,128]. This infiltrate is most commonly perivascular, in the mid-dermis, or at the dermal-epidermal junction. Other common findings include dermal edema, vascular inflammation with nuclear dust, endothelial cell prominence, and red blood cell extravasation, and occasionally true leukocytoclastic vasculitis [15,43,49,118,127,128]. Despite the high frequency of peripheral hypereosinophilia, only a minority of patients show eosinophilic infiltrates on histopathology [126].

The histology of lymph nodes in DReSS generally show a pseudo-lymphoma pattern or similar appearance to a viral infection [1,4,129]. The liver pathology in patients with severe hepatic involvement shows intralobular necrosis, CD8+-activated lymphoid infiltrates, Kupffer cell hyperplasia with erythrophagocytosis, and an inconsistent presence of eosinophils [31]. A recent systematic review of cardiac involvement in DReSS showed fulminant eosinophilic myocarditis with acute necrosis in 70% of cardiac biopsies [115]. Biopsies of other organs involved in DReSS are more rarely reported on, however renal biopsies (when performed) often show patterns consistent with tubulointerstitial nephritis [130,131,132].

## 8. Differential Diagnosis

There are many conditions that mimic DReSS, including viral infections (EBV, SARS-CoV-2, CMV, and HIV), bacterial sepsis, toxic shock syndrome, erythroderma from primary dermatological conditions (psoriasis, eczema), Kawasaki’s, Still’s disease, lymphoma, mycosis fungoides, hypereosinophilic syndrome, connective tissue diseases, hemophagocytic syndrome, and angioimmunoblastic lymphadenopathy [1,3,133,134]. The other SCARs (SJS/TEN and AGEP) must be considered as well, particularly in the early stages of disease. MPE is high on the differential but is distinguished based on the degree of systemic involvement.

Compared to DReSS, the systemic symptoms of SJS/TEN (often liver or lung) are less frequent and often milder [135]. Furthermore, the cutaneous findings are typically more severe with skin necrosis, extensive sloughing, and severe hemorrhagic mucosal disease [3,136]. Although exfoliative dermatitis and desquamation of the skin can be present in DReSS, SJS/TEN is distinct in showing full thickness epidermal necrosis. Blistering in DReSS can occur from dermal edema but are typically tense compared to the flaccid bullae of SJS/TEN [135]. AGEP is characterized by multiple pinpoint sterile non-follicular pustules often in the intertriginous areas [137]. Comparatively, DReSS may have a pustular component but lacks predilection for the folds. Furthermore, DReSS typically follows a more delayed onset and protracted course compared to SJS/TEN and AGEP. Overlap syndromes can occur but are rare, occurring in 2.1% of all SCARs in one 2012 retrospective study [138]. Hypereosinophilic syndrome can mimic DReSS with its eosinophilia and cutaneous involvement, however liver involvement is much less frequent and more often demonstrates cardiac and neurologic impairment [139].

## 9. Prognosis and Long-Term Outcomes

The mortality rate in DReSS is frequently quoted at 10% [43,107,109]. More recent studies have found lower estimates ranging from 1.7% to 8.8% [15,16,28,33]. Mortality in the pediatric population is reported to be 5.4% [19]. The most common causes of death are hepatic failure, multiorgan failure and sepsis [4,43,63,140]. Poor prognostic factors for DReSS include pancytopenia, older age, CMV reactivation, allopurinol or minocycline-induced DReSS, and renal and hepatic involvement [4,6,27,43]. Cardiac involvement has also been associated with higher mortality rates, up to 45% in a recent review [115,141]. Recently, Mizukawa et al. [17] created a composite score for evaluating DReSS severity based on a variety of factors including age, allopurinol-exposure, the need for pulsed prednisone, the duration of drug exposure after symptom onset, fever duration, % BSA, appetite loss, liver involvement, renal dysfunction, and C-reactive protein (CRP). They found higher scores (≥4) were associated with CMV reactivation and CMV-related complications, higher steroids doses, longer hospitalizations, and higher mortality rates.

Signs and symptoms of DReSS may persist for weeks after withdrawal of the culprit drug, with a mean recovery time around 6–9 weeks [27]. Furthermore, there have been multiple reports of patients developing autoimmune sequelae after DReSS including Hashimoto’s thyroiditis, Grave’s disease, fulminant type 1 diabetes, systemic lupus erythematosus, alopecia areata, vitiligo, autoimmune hemolytic anemia, thrombotic thrombocytopenic purpura and rheumatoid arthritis [35,60,61,113,142]. Chen et al. [142] found the cumulative incidence of long-term sequelae to be 11.5% (both immune and non-immune). Multiple studies have found autoimmune thyroid disease and fulminant type 1 diabetes to be the most common autoimmune sequelae, with onset typically 2–4 months post-DReSS [61,113,142,143]. Functional deficiency of Tregs upon clinical resolution of DReSS has been put forth as one explanation for the autoimmune sequelae seen in these patients [34,113]. Other non-immune long-term sequelae include end-stage renal disease requiring hemodialysis [33,109,124]. Multiple drug hypersensitivity syndrome (MDH), defined as an immune mediated hypersensitivity reaction to two or more unrelated drugs confirmed by skin or by in vitro testing, is a recently recognized entity that may occur after DReSS [144]. In a study of 87 patients with DReSS, 40% experienced relapse after exposure to drugs different from their original causative agent, with 17% overall meeting criteria for MDH [144].

## 10. Treatment

The mainstay treatment of DReSS remains systemic steroids alongside identification and immediate withdrawal the culprit drug [3,30,47,145]. All patients should be hospitalized, at least in the initial phase, to monitor for delayed systemic involvement and response to treatment. The initial assessment should include a complete blood cell count with a differential, peripheral blood smear, liver enzymes and liver function tests, kidney function with creatinine and urea, electrolytes, pancreatic enzymes including lipase and amylase, and viral serology for HHV-6 [47,145]. Further viral testing and laboratory work-up can be done as clinically indicated based on history, physical exam, and differential diagnosis. Supportive care with close monitoring, fluid and electrolyte replacement, hemodynamic support and adequate skin care is also imperative [47]. Once a culprit drug is identified, all drugs in that class and possible cross-reactive medications (e.g., all aromatic antiepileptics in the context of aromatic antiepileptic associated DReSS) should be avoided. It should be clearly communicated to the patient that the causative drug needs to be avoided indefinitely. Furthermore, all family members with first-degree blood relativity should be counselled on avoiding the drug, at least until more widespread testing for genetic susceptibility becomes available to aid in clarifying the risk to family members [146].

Based on expert consensus, the Spanish Guidelines propose a stepwise treatment algorithm depending on the degree of organ involvement [47]. In non-serious DReSS with no systemic involvement or only stage I drug-induced liver injury or stage I kidney injury, they suggest high potency topical steroids alone. For DReSS with more severe organ involvement, oral prednisone at a dose ranging from 0.5–1 mg/kg/day is suggested with gradual taper over 4–6 weeks or longer. If a relapse occurs during steroid tapering, a more gradual taper is suggested or employing steroid-sparing agents. If control is not obtained with steroids, cyclosporine, or less evidence-based therapies such as intravenous immunoglobulin (IVIG), plasmapheresis or cyclophosphamide can be used. If viral reactivation and life-threatening signs occur, or viral reactivation is suspected of contributing to severe complications such as encephalitis, hemophagocytosis, or severe erosive colitis, adding an antiviral such as IV ganciclovir or oral valganciclovir is suggested.

The French Society of Dermatology have also published a management protocol based on four visceral involvement severity categories [145]. If there is minimal systemic involvement, topical corticosteroids, emollients, and antihistamines can be used. For severe involvement (transaminase levels ≥ 5 times normal, renal involvement, pneumonia, hemophagocytosis or cardiac involvement) they suggest systemic corticosteroids equivalent to 1 mg/kg/day of prednisone and multidisciplinary evaluation. For life threatening signs including bone marrow failure, encephalitis, severe hepatitis, renal failure, and respiratory failure, they recommend systemic steroids with intravenous immunoglobulin (IVIG) at a dose of 2 g/kg over five days. Finally, for major viral reactivation they encourage combining antivirals with steroids and/or IVIG.

It is important to note that DReSS guidelines are largely based on expert opinion with no randomized control trials and only a few prospective studies. While these protocols suggest topical steroids alone if there is minimal systemic involvement, it is critical to understand that DReSS may progress over time to have more severe and widespread involvement. Therefore, frequent re-assessment is imperative, alongside the initiation of systemic steroids if progression is noted. Importantly, skin involvement is not indicative of systemic progression and treatment adjustments should not be based on cutaneous assessment alone [147]. Furthermore, flares are common during DReSS even while on systemic therapy, and do not indicate that a treatment is ineffective or should be stopped. There is consensus that steroids should be administered for a long period of time with a slow taper, ranging from 6 weeks to 3 months or longer if clinically indicated [31,35,63,109]. Many studies have shown that relapses tend to occur when tapered too fast [30,31,36].

Some studies have suggested that patients treated with topical steroids have better outcomes, fewer complications, and less viral reactivation compared to those treated with systemic steroids [148,149,150]. However, this conclusion is challenging to interpret as patients treated with topical agents often have less severe DReSS to begin with. Funck-Brentano et al. [148] examined 38 patients with DReSS and found that most patients could be managed with topical steroids alone, with systemic steroids reserved for life-threatening organ involvement only. However, one death did occur in the topical steroid group compared to no deaths in the systemic steroid group. Brin et al. [149] investigated the outcome of switching patients with mild/moderate disease from systemic to topical steroids. They found an unfavorable outcome in 25% of patients, indicating topical steroids alone may not be sufficient for all patients with moderate disease. Regarding DReSS with isolated liver involvement, reports on systemic steroid efficacy have been mixed. Some reports have shown a clear benefit, while several others have shown no difference in mortality or the need for transplant [31,151].

There is little evidence regarding alternative treatments for DReSS. Case reports and small studies have examined cyclosporine, IVIG, mycophenolate mofetil, cyclophosphamide, and rituximab as possible alternate therapeutic options. In a study by Nguyen et al. [152] cyclosporine was associated with shorter treatment duration, reduced disease progression, improved clinical and laboratory markers, and a shorter hospital stay compared to treatment with steroids. Multiple case reports have also shown efficacy of short courses of cyclosporine in steroid-refractory DReSS, while others have shown no improvement in this context [3,153,154]. Although reports of successful treatment with IVIG have been reported, IVIG is not recommended as monotherapy in DReSS based on results of a small study showing frequent adverse events after treatment—it may be a useful adjunctive therapy in addition to steroids to help replenish low immunoglobulin levels [155]. Mycophenolate mofetil, cyclophosphamide, rituximab, and N-acetylcysteine have also been shown to be effective in a several cases reports; however more studies are needed to explore their efficacy and safety in this condition [3].

Because of the prolonged course of DReSS, high occurrence of relapse, and potential long-term sequelae, it is crucial that patients have a long term follow up outside of the hospital, specifically monitoring for the development of autoimmune disease and/or subsequent drug reactions.

## 11. Limitations

The main limitation of the literature on DReSS is that the evidence is predominantly based on retrospective studies and case reports. Moreover, as there are no double-blind, placebo-controlled, randomized-control trials, treatment recommendations are largely based on clinical opinion. Secondly, the nomenclature surrounding this topic is complex and diverse, often creating un-necessary confusion both clinically and within research realms. Thirdly, different ethnic regions not only affect genetic predisposition to DReSS, but also impact the recognition of the disease and the clinical manifestations, making it challenging to characterize on a global level. Finally, this summary is not a systematic review, which limits some degree of objectivity. However, careful attention was paid to present multiple different perspectives on the heavily debated topics in DReSS.

## 12. Conclusions

There is ongoing knowledge gathering on the topic of DReSS, a heterogenous clinical syndrome. This review aimed to present the most updated information on the epidemiology, pathogenesis, risk factors, clinical features, diagnostics, prognosis, and treatment of DReSS. While significant advances have been made, more studies are needed to better characterize its etiology, predisposing factors, effective treatments, and long-term outcomes. As more definitive information is collected regarding topics such as the role of viral reactivation and immune-dysregulation, non-drug triggers, predisposing risk factors, and variable hematologic and internal organ involvement, the authors propose that a change in the nomenclature may be indicated in the future to better reflect the true nature of this condition known as DReSS.

## Figures and Tables

**Table 1:** Drugs associated with DReSS.

Drug Category	Drug Name
Anticonvulsant	Carbamazepine *, lamotrigine *, phenobarbital *, levetiracetam *, valproate, phenytoin *, oxcarbazepine, ethosuximide, zonisamide, gabapentin
Anti-infective	Vancomycin *, minocycline *, ampicillin/amoxicillin, ampicillin/sulbactam, amoxicillin-clavulanic acid, cefadroxil, cefepime, cefixime, cefotaxime, ceftazidime, imipenem, meropenem, piperacillin/tazobactam * metronidazole, linezolid, azithromycin, levofloxacin, benznidazole *, clindamycin, hydroxychloroquine, teicoplanin, voriconazole
Anti-tuberculosis	Rifampin *, isoniazid, pyrazinamide, streptomycin, ethambutol
Anti-viral	Abacavir *, nevirapine *, boceprevir, telaprevir, and zalcitabine
Antidepressant and antipsychotic	Bupropion, fluoxetine, olanzapine, amitriptyline, clomipramine
Sulfonamide	Trimethoprim-sulfamethoxazole *, dapsone *, sulfasalazine *, salazosulfapyridine *, furosemide
Antineoplastic and immunomodulators	Sorafenib, vismodegib, vemurafenib, efalizumab and imatinib, azathioprine, chlorambucil, leflunomide, lenalidomide
Antihypertensive	Amlodipine, captopril, diltiazem, spironolactone
Analgesics	Diclofenac, celecoxib, ibuprofen, aspirin, metamizole, phenylbutazone, dexketoprofen, codeine phosphate
Miscellaneous	Allopurinol *, atorvastatin, Traditional Chinese Medicine (see Wang and Mei for specific products [46]), quinine, mexiletine, omeprazole, esomeprazole, strontium ranelate, epoetin alfa, ranitidine, thiamine, cyanamide, vitamin B12, sitagliptin, tribenoside, iodinated contrast media, rivaroxaban

* Drugs most often associated with DReSS. Modified from Martinez et al. [3], Cabanas et al. [47], James et al. [48], Cacoub et al. [27].

**Table 3:** Diagnostic criteria for DiHS established by Japanese consensus group [21].

Diagnostic Criteria
1. Maculopapular rash developing > 3 weeks after starting a limited number of drugs
2. Prolonged clinical symptoms after discontinuation of the causative drug
3. Fever (>38 °C)
4. Liver abnormalities (ALT > 100 U/L) or other organ involvement
5. Leukocyte abnormalities (at least one present) - Leukocytosis (>11 × 10^9^/L) - Atypical lymphocytosis (>5%) - Eosinophilia (>1.5 × 10^9^/L)
6. Lymphadenopathy
7. HHV-6 reactivation
The diagnosis is confirmed by presence of all seven criteria above (typical DiHS) or five of seven criteria (atypical DiHS)

**Table 4:** RegiSCAR validation score for DReSS syndrome [5].

RegiSCAR Criteria	Score					
	−1	0	1	2	Min	Max
Fever ≥ 38.5 °C	N/U	Y			−1	0
Enlarged lymph nodes) (>1 cm size, at least 2 sites)		N/U	Y		0	1
Eosinophilia		N/U	700–1499/μL (10–19.9% if leukopenia)	≥1500/μL (≥20% if leukopenia)	0	2
Atypical lymphocytes		N/U	Y		0	1
Skin involvement					−2	2
- Body surface area ≥ 50%		N/U	Y			
- Rash consistent with DReSS	N	U	Y			
- Biopsy consistent with DReSS	N	Y/U				
Organ involvement -liver, kidney, lung, muscle/heart, pancreas, and other organ(s)		N/U	Y/Y/Y/Y/Y/Y		0	2
Resolution ≥ 15 days	N/U	Y			−1	0
Evaluation other potential causes (ANA, blood culture, serology for HAV/HBV/HCV, Chlamydia, Mycoplasma pneumoniae) If none positive and ≥3 negative		Y			0	1
Total score	<2, Excluded; 2–3, Possible; 4–5, Probable; >5, Definite				−4	9

N, no; Y, yes; U, unknown. See Kardaun et al. [5] for further details on specific scoring criteria.
